# Effect and mechanism of mixed fungi synergistically enhancing the leaching of elements from wollastonite

**DOI:** 10.1128/aem.02519-25

**Published:** 2026-07-21

**Authors:** Chengbing Chang, Jianying Guo, Shengyu Liu, Quanbao Wen

**Affiliations:** 1 College of Mining Engineering, Taiyuan University of Technology 47846, Taiyuan, Shanxi, China; 2 Key Laboratory of In-situ Property-improving Mining of Ministry of Education, Taiyuan University of Technology 47846, Taiyuan, Shanxi, China; Chalmers tekniska hogskola AB, Gothenburg, Sweden

**Keywords:** wollastonite, mixed culture, *Ramichloridium apiculatum*, *Talaromyces pseudofuniculosus*, metabolic products, bioleaching mechanism

## Abstract

The tight encapsulation of Ca^2+^ in wollastonite by silicon-oxygen tetrahedra makes it difficult to leach, restricting the carbonation reaction rate. Microbial leaching technology offers distinct advantages for enhancing the release of carbon-fixing elements due to its mild operating conditions and low cost. While single-strain weathering of wollastonite has been thoroughly investigated, the effectiveness and mechanisms of mixed strains remain unclear. This study systematically investigated their dissolution patterns on wollastonite using a mixed culture of *Ramichloridium apiculatum* CMS-1 and *Talaromyces pseudofuniculosus* hzt-3′ in different ratios. The synergistic leaching mechanism of the mixed consortium was revealed through a comprehensive analysis of fermentation broth dynamics, residue transformation, and the expression of key metabolites, including organic and amino acids. The results demonstrated significantly superior Ca^2+^ and Si^4+^ leaching by mixed strains compared to pure cultures. The 20% CMS-1 + 80% hzt-3′ ratio achieved leaching efficiency of 79.95% ± 6.37% for Ca^2+^ and 18.59% ± 0.11% for Si^4+^, representing an approximately 30.17-fold increase over CMS-1 pure culture (2.65% Ca^2+^ leaching efficiency). Metabolic analysis showed accelerated polysaccharide consumption, increased extracellular proteins, and significantly elevated organic acid and amino acid production in mixed culture. The strain CMS-1 primarily produced oxalic acid (0.66 ± 0.033 mg/mL), while strain hzt-3′ mainly generated gluconic acid (1.60 ± 0.0798 mg/mL). In mixed culture, CMS-1 synthesized extracellular polysaccharides and proteins that were utilized by hzt-3′, which upregulated the production of gluconic acid, isocitric acid, L-glutamine, and L-tyrosine, collectively enhancing acidity and chemical dissolution. Additionally, surface cracks and grooves on leaching residues indicated additional biophysical disruption.

**IMPORTANCE:**

This study elucidates the synergistic mechanism of a mixed microbial consortium for silicate weathering, a key process for enhanced mineral carbonation. It reveals how cross-feeding interactions between fungi upregulate organic acid production, dramatically accelerating wollastonite dissolution compared to single strains. The findings provide a novel and efficient biological strategy to overcome the intrinsic recalcitrance of silicate minerals, advancing the development of low-cost, environmentally friendly technologies for carbon dioxide sequestration through enhanced mineral carbonation.

## INTRODUCTION

The rapid increase in the concentration of greenhouse gases such as CO_2_ in the atmosphere has led to global warming, posing severe challenges to both the Earth’s ecosystems and human society. Reducing and stabilizing atmospheric CO_₂_ concentrations has become one of the most urgent global tasks (1–4). Carbon capture, utilization, and storage technology is widely regarded as an indispensable part of the technological portfolio for achieving carbon neutrality (5, 6). Among these, CO_2_ mineral sequestration technology utilizes the reaction between carbon dioxide and calcium-magnesium silicate minerals to permanently store CO_2_ in the form of carbonates (7). Its scientific principles stem from the naturally slow weathering process of silicate rocks, offering greater stability and safety compared to methods such as oceanic or geological storage (8, 9). As a result, it is increasingly attracting researchers’ attention.

Silicate minerals on Earth (such as olivine [10], serpentine [11], wollastonite [12], and anorthite [13]) are extremely abundant and theoretically capable of sequestering centuries’ worth of global CO_2_ emissions (11). Among the numerous silicate minerals, wollastonite is particularly favored due to its specific physical and chemical properties. Compared to other silicate minerals, wollastonite has a relatively simple crystal structure and exhibits slightly faster reaction kinetics under mild conditions (12). Furthermore, the products of its carbonation reaction are calcium carbonate and silicon dioxide, both of which are environmentally friendly and widely useful materials (14). However, during the carbonation process, the calcium within the crystal structure of wollastonite is tightly surrounded by silicon-oxygen tetrahedra, making it difficult to release Ca^2+^ to react with CO_2_; this significantly limits the carbonation reaction rate and conversion efficiency (15). To overcome the reaction kinetic barriers, the mineral or the reaction process must undergo “activation” treatment. Current common pretreatment methods for wollastonite include mechanical grinding (16), thermal activation (17, 18), and chemical activation (18, 19). Although these methods can achieve relatively high conversion rates, they are often accompanied by extremely high energy consumption and substantial costs, which may have more associated carbon dioxide release than the liberated calcium can sequestrate, defying the point of the entire process. Therefore, actively seeking new activation methods characterized by low pollution, low energy consumption, and low cost has become an imperative for enhancing the carbon sequestration process of wollastonite.

Microbial mineral leaching offers a relatively mild and low-cost approach to modify mineral structures and release key cations, thereby significantly overcoming the kinetic limitations in the carbonation process and enhancing the overall efficiency and feasibility (20, 21). Current research on the interaction between microorganisms and wollastonite, both domestically and internationally, remains relatively limited. Only Chiang et al. (20) demonstrated that bacterial and fungal leaching positively increased the solubility of wollastonite, altered alkalinity, mineralogy, and the morphology of solid materials. Salek et al. (22) investigated the kinetic processes when wollastonite was co-cultured with anaerobic fermentative bacteria, determining that the dissolution rate of wollastonite is primarily governed by the amount of organic acids produced. Heavy metal ions can inhibit microbial growth (23). Mardin et al. (24) found that when the ZnO content in wollastonite reached 31.55%, it inhibited the growth of a filamentous pathogenic fungus. Pokrovsky et al. (25) treated wollastonite with the soil bacterium *Pseudomonas aureofaciens* and observed that the dissolution rates of calcium and silicon were 20% higher in the live bacterial culture group compared to the dead bacteria control group. Furthermore, some studies have clarified the role of bioenzymes in the weathering of wollastonite by *Aspergillus niger* and *Aspergillus nidulans* from the perspective of gene expression (26, 27). These studies all involve the pure culture of wollastonite with single strains. To date, research on treating wollastonite with mixed strains remains a blank. Opting for mixed strains to activate wollastonite essentially simulates the natural scenario where microorganisms participate in mineral weathering in community forms. By constructing a functionally diverse and mutually supportive microbial consortium, the activation of wollastonite and the leaching of calcium can be achieved more efficiently, stably, and economically, laying a solid foundation for subsequent mineral carbonation and CO_2_ sequestration.

This study aims to address the research gap in wollastonite dissolution under mixed microbial strains. Two fungal strains, *Ramichloridium apiculatum* CMS-1 and *Talaromyces pseudofuniculosus* hzt-3′, isolated from mineral-rich soil, were selected as representative members of natural mineral-weathering communities. A mixed-culture system better simulates the multi-species interaction scenario present in real bioleaching environments, making the research findings more ecologically relevant and applicable. Preliminary screening and bioleaching experiments revealed that both individual strains exhibit effective bioleaching performance on silicate minerals (28, 29).

Based on this, the study systematically investigates the leaching efficiency and patterns of ions (Ca^2+^ and Si^4+^) from wollastonite in both pure-culture and mixed-culture systems. Through measurements of organic acids, amino acids, extracellular polysaccharides, and protein content, the feedback effect of wollastonite leaching on the metabolic activities of the mixed strains was elucidated. Furthermore, by combining analytical results from Fourier transform infrared (FT-IR), X-ray diffractometer (XRD), and scanning electron microscopy-energy-dispersive X-ray spectroscopy (SEM-EDS), the synergistic mechanism of the mixed strains in dissolving wollastonite was clarified. The research findings provide a theoretical basis for the application of synergistic microbial communities in enhancing CO_2_ sequestration using silicate minerals.

## MATERIALS AND METHODS

### Experimental material

The wollastonite sample powder was purchased from Guzhang Shan Lin Shi Yu Mineral Co., Ltd. Its chemical composition was determined by X-ray fluorescence spectrometry (XRF), and the results are shown in Table 1. The main chemical components of the wollastonite are CaO (56.69%) and SiO_2_ (40.23%), with MgO content accounting for only 0.78%. Therefore, the magnesium content in wollastonite can be considered negligible during ion concentration measurements.

**TABLE 1:** Chemical composition of wollastonite (wt%)

CaO	SiO_2_	Al_2_O_3_	MgO	Fe_2_O_3_	Na_2_O	MnO	K_2_O	P_2_O_5_	V_2_O_5_	Others
56.69	40.23	0.87	0.78	0.64	0.31	0.14	0.13	0.11	0.06	0.04

**Fig 1:**
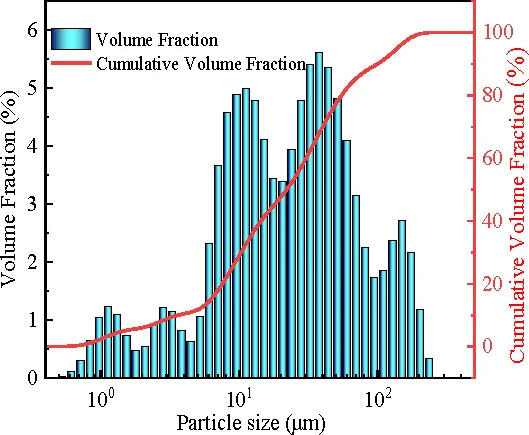
The particle size distribution curve of the wollastonite powder.

The particle size distribution curve of the wollastonite powder is shown in Fig. 1. In the figure, *d*_10_, *d*_50_, and *d*_90_ represent the particle sizes corresponding to 10%, 50%, and 90% of the cumulative volume fraction, respectively. It can be observed that the wollastonite exhibits a broad particle size distribution range, spanning from the sub-micron level (0.1 μm) to the ultra-fine particle level (100 μm). The sample displays significant polydispersity. Within this size range, distinct bimodal and multimodal phenomena are observed, indicating the multi-scale structural characteristics of the wollastonite sample during the crushing process (30). The *d*_10_, *d*_50_, and *d*_90_ values for the wollastonite are 3.83, 21.56, and 110.16 μm, respectively.

### Preparation of fungal spore suspension

**Fig 2:**
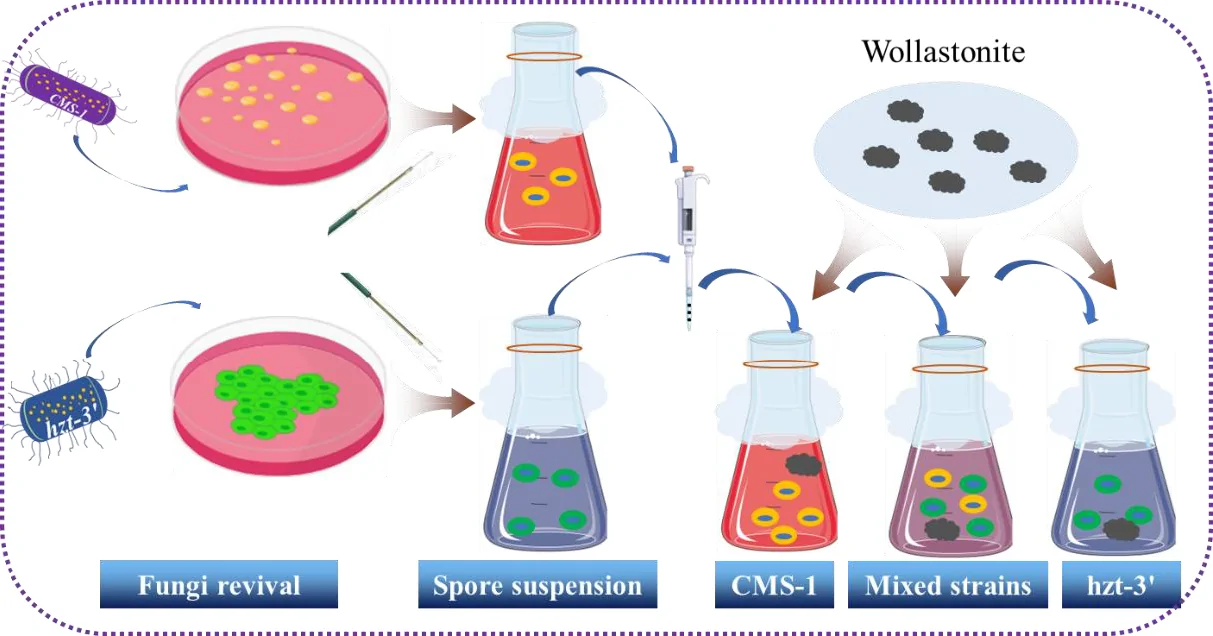
Experimental flowchart.

The strains *Ramichloridium apiculatum* CMS-1 and *Talaromyces pseudofuniculosus* hzt-3′ were, respectively, inoculated onto Czapek solid medium (sucrose, 30 g; NaNO_3_, 3 g; K_2_HPO_4_·3H_2_O, 1 g; KCl, 0.5 g; MgSO_4_·7H_2_O, 0.5 g; FeSO_4_·7H_2_O, 0.01 g; agar, 20 g; and deionized water, 1,000 mL [pH 7.5]) and activated by culturing at 30°C until robust mycelial growth was observed. The surface of the medium was rinsed with a sterile 0.1% Tween 80 solution, and the mycelia on the medium surface were gently scraped off using an inoculation loop and transferred to a conical flask. The flask was shaken for 2 h, and the mycelia were filtered through sterile absorbent cotton. The resulting spore suspension was diluted to a concentration of 10^6^ spores/mL for subsequent use (31, 32). The phylogenetic trees of the two fungal strains are shown in Fig. S1 and S2, and the preparation process is illustrated in Fig. 2.

### Microbial leaching experiment

A 100 mL volume of modified Czapek liquid medium (sucrose, 30 g; NaNO_3_, 3 g; K_2_HPO_4_·3H_2_O, 1 g; KCl, 0.5 g; and deionized water, 1,000 mL [pH 7.5]) was poured into a 250 mL conical flask, and 0.5 g of the wollastonite sample was added. The conical flask was then placed in an autoclave and sterilized at 121°C for 20 min. After cooling to room temperature, 1 mL of mixed fungal strains was inoculated according to the specified proportions. Finally, the flask was placed in a constant-temperature incubator shaker at 30°C and 150 rpm for cultivation. Samples were taken for measurement every 2 days, with each experimental group repeated three times.

The mixed-strain proportions were set to six ratios: 100% CMS-1, 80% CMS-1 + 20% hzt-3′, 60% CMS-1 + 40% hzt-3′, 40% CMS-1 + 60% hzt-3′, 20% CMS-1 + 80% hzt-3′, and 100% hzt-3′. The design of the leaching experiment is shown in Fig. 2.

### Determination of liquid phase parameters

#### Polysaccharide content

The polysaccharide content was determined using the sulfuric acid-phenol method (33). An adequate amount of glucose was dried in a constant temperature blast drying oven at 70°C for 1 h. Precisely 10.00 mg of the dried glucose was weighed, dissolved in distilled water, and diluted to a final volume of 100 mL in a volumetric flask to prepare a 0.1 g/L standard glucose solution. For the 6% phenol solution, phenol was dissolved in a 60°C water bath because it is solid at room temperature. Then, 6 mL of the liquefied phenol was taken and diluted with distilled water to a final volume of 100 mL. Concentrated sulfuric acid was added to glucose solutions of different concentration gradients, and the mixtures were thoroughly mixed. After standing for 30 min to cool to room temperature, the absorbance of the glucose solutions at different concentrations was measured at a wavelength of 490 nm using an ultraviolet spectrophotometer. The standard curve for polysaccharides was plotted as *y* = 12.5057*x* + 0.0235 (*R*^2^ = 0.9901), and the results are shown in Fig. S3. The procedure for determining the polysaccharide content in the samples was consistent with that used for the standard curve.

#### Protein content

The protein concentration in the leaching suspension was determined using the commercially available “Bradford Protein Assay Kit” (Product No. PH0325) (34). A 100 μL aliquot of the protein standard (bovine serum albumin) was mixed with 2.4 mL of diluent (phosphate-buffered saline) to prepare a diluted solution with a final concentration of 0.2 mg/mL. Then, 10 mL of 5× G250 dye reagent was combined with 40 mL of distilled water and mixed thoroughly to prepare the 1× G250 dye working solution. The dye was added to the standard protein solutions, and the mixtures were inverted three to five times to ensure homogeneity. After a 3–5 min reaction period, the absorbance of the solutions at different concentrations was measured at a wavelength of 595 nm. A standard protein curve was plotted as *y* = 0.0208*x* + 0.0082 (*R*^2^ = 0.9955), and the results are shown in Fig. S4.

For the test samples, a 500 μL aliquot of the sample was accurately pipetted into a test tube, followed by the addition of 5 mL of 1× G250 dye working solution. After reacting for 3–5 min, the absorbance was measured at a wavelength of 595 nm, and the protein content in the sample was calculated accordingly.

#### Determination of organic acids and amino acids

The content of organic acids and amino acids in the metabolic products was determined using liquid chromatography electrospray ionization mass spectrometry (LC-ESI-MS/MS) (UHPLC-Qtrap). The specific parameter settings were as follows.

##### Chromatographic conditions for organic acid determination

An ExionLC AD system with a Waters HSS T3 column (2.1 × 150 mm, 1.8 μm) was used; column temperature, 40°C; injection volume, 2 μL; mobile phase A, 0.03% formic acid in water; and mobile phase B, 0.03% formic acid in methanol.

##### Mass spectrometric conditions for organic acid determination

A SCIEX QTRAP 6500+ was used for negative ion mode detection; Curtain Gas (CUR), 35; Collision Gas (CAD), Medium; Ion Spray Voltage (IS), −4,500 V; Temperature (TEM), 500°C; Ion Source Gas 1 (GS1), 55; and Ion Source Gas 2 (GS2), 55.

##### Chromatographic conditions for amino acid determination

An AdvanceBio MS Spent Media column (2.1 × 50 mm, 2.7 μm) was used; column temperature, 40°C; injection volume, 1 μL; mobile phase A, 0.1% formic acid and 10 mM ammonium formate in 95% aqueous solution; and mobile phase B, 0.1% formic acid and 10 mM ammonium formate in 95% acetonitrile solution.

##### Mass spectrometric conditions for amino acid determination

A SCIEX QTRAP 6500+ was used for positive/negative ion mode detection; CUR, 35; CAD, Medium; IS, +5,500 V/−4,500 V; TEM, 550°C; GS1, 50; and GS2, 50. Scheduled MRM, basic, with an MRM detection window of 120 s.

### Ion concentration determination

The calcium ion concentration in the solution was determined by EDTA titration (35), and the silicon ion content was measured using the silicon molybdenum blue spectrophotometric method (36).

The ion leaching efficiency was calculated as follows:


(1) $$E=\frac{c_{1}V}{\omega m}\times 100,$$

where *c*_₁_ is the element concentration in the solution (g/L), *V* is the solution volume (L), *ω* is the element content in the raw ore (%), and *m* is the initial mass of the solid (g).

### Characterization methods

The chemical composition of wollastonite was determined using a PANalytical Axios XRF spectrometer.

Surface functional group changes were analyzed using a TENSOR 27 FT-IR spectrometer. The samples were mixed with potassium bromide (KBr) at a mass ratio of 1 mg:100 mg, and the spectra were recorded in the wavenumber range of 4,000–500 cm^−1^.

Phase changes before and after leaching were analyzed using a MiniFlex600 XRD with Cu Kα radiation. The operating conditions were as follows: tube voltage, 40 kV; tube current, 15 mA; divergent slit, 1.25°; scattering slit, 1.25°; and receiving slit, 0.3 mm. The scanning range was 2θ = 5°–85° at a scan rate of 12°/min. The interplanar spacing was calculated using Bragg’s law (37), as shown in equation 2:


(2) $$d=\frac{n\lambda }{2sin\theta },$$

where *n* is the diffraction order, *λ* is the incident X-ray wavelength (1.5406 Å), *θ* is the angle between the incident X-ray direction and the crystal planes (°), and *d* is the perpendicular distance between two adjacent parallel crystal planes (Å).

Sample morphology and energy-dispersive X-ray spectroscopy mapping were performed using a TESCAN MIRA3 field-emission scanning electron microscope. An accelerating voltage of 3 kV was used for morphological imaging, while 15 kV was applied for EDS analysis.

X-ray photoelectron spectroscopy (XPS, Thermo Scientific K-Alpha) was used to analyze the chemical states of elements. The analysis chamber operated at a vacuum of approximately 5 × 10^−9^ mbar. Monochromatic Al Kα radiation (energy: 1,486.6 eV) was used as the excitation source, with an accelerating voltage of 12 kV, a filament current of 6 mA, and the analyzer operating in constant analyzer energy mode.

## RESULTS AND DISCUSSION

### Leaching behavior of elements from minerals

**Fig 3:**
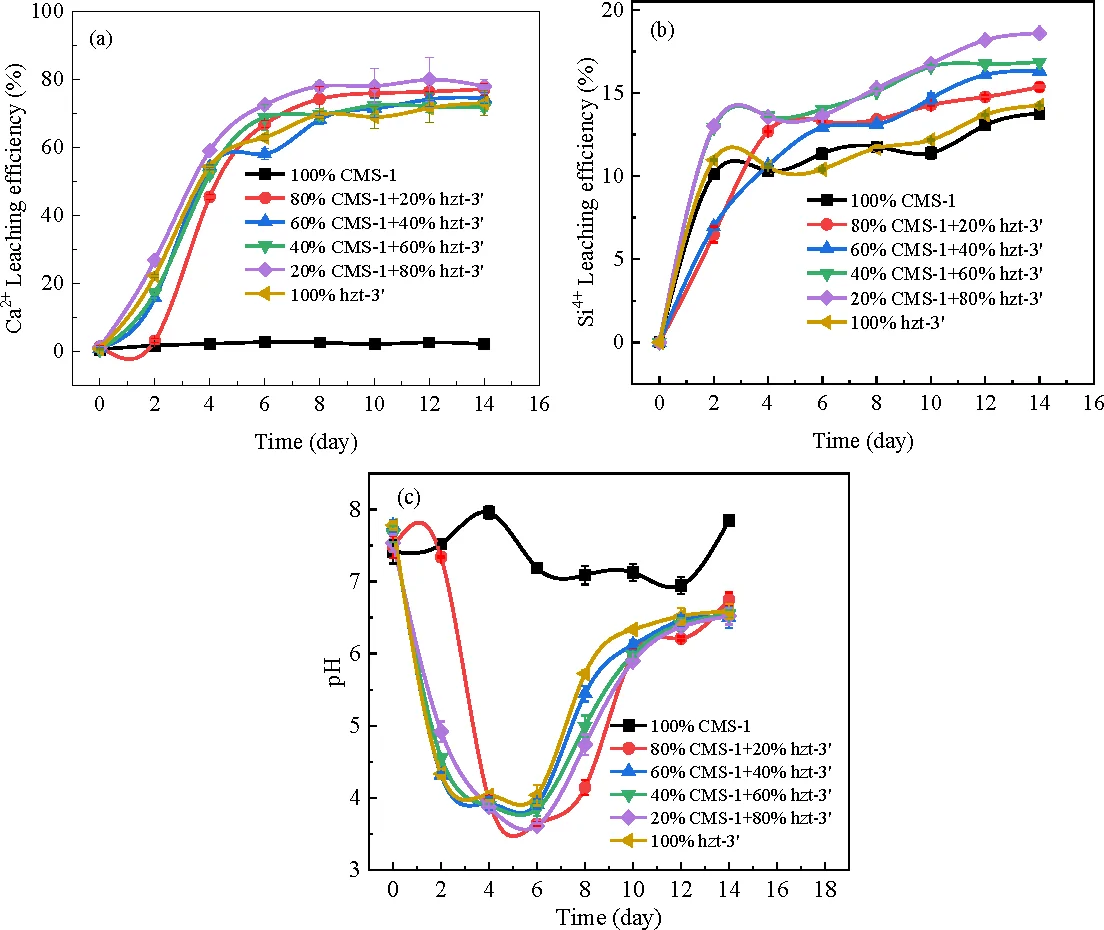
Variation trends of (**a**) Ca^2+^, (**b**) Si^4+^, and (**c**) pH value during the bioleaching of wollastonite by mixed fungal strains.

The leaching behavior of Ca^2+^ from wollastonite under the action of mixed fungi is shown in Fig. 3a. The leaching efficiency of Ca^2+^ in all culture systems increased significantly with the extension of cultivation time (0–14 days), exhibiting typical microbial leaching kinetics: the leaching efficiency rose sharply during the early stage (0–6 days), while the growth rate slowed markedly in the later stage (6–14 days), gradually leveling off and reaching a leaching plateau. This kinetic pattern indicated that the leaching process is primarily driven by microbial acid production in the early stage, while it may become limited in the later stage by factors such as the reduction of reactive sites on the mineral surface or the accumulation of metabolic products (38, 39). The Ca^2+^ leaching efficiency in the 20% CMS-1 + 80% hzt-3′ system was consistently higher than in other systems throughout the leaching process. At 12 days, the leaching rate reached 79.95% ± 6.37%, which was 30.17 times and 1.11 times higher than that in the single-strain systems of CMS-1 and hzt-3′, respectively. The poor leaching efficiency of wollastonite in the 100% CMS-1 system is attributed to silicate ions (SiO_3_^2−^) capturing protons (H^+^) from water, forming hydrogen silicate ions (HSiO_3_^−^) and hydroxide ions (OH^−^), which results in an alkaline solution (40). Since strain CMS-1 can only produce a small amount of acid, the metabolically generated acid preferentially neutralizes the hydroxide ions (OH^−^), thereby making it difficult to leach Ca^2+^, whose leaching mechanism is dominated by proton exchange (41).

Figure 3b shows the leaching results of Si^4+^ from wollastonite under the action of mixed fungi. The kinetic process of Si^4+^ leaching can be divided into two main stages. Rapid leaching stage (0–4 days): the leaching efficiency increased sharply in all systems during this stage, indicating vigorous microbial metabolic activity and the fastest rate of destruction and dissolution of the wollastonite structure. Decelerated equilibrium stage (4–14 days): the growth rate of leaching efficiency slowed significantly and gradually stabilized. This may be attributed to the depletion of readily reactive components in the mineral, the passivation of leaching products on the mineral surface, or a decline in microbial activity due to nutrient depletion. The Si^4+^ leaching efficiency in the mixed fungal culture systems was significantly higher than that in the single-strain systems. Among them, the 20% CMS-1 + 80% hzt-3′ system exhibited the best leaching performance, reaching 18.59% ± 0.11% at 12 days, which was 1.42 times and 1.30 times higher than that in the 100% CMS-1 and 100% hzt-3′ systems, respectively.

Comparing the leaching results of Ca^2+^ and Si^4+^ from wollastonite in the 100% CMS-1 system reveals a significant difference: the Ca^2+^ leaching efficiency was only 2.46%, while the Si^4+^ leaching efficiency reached 13.70%. This notable discrepancy arises from their distinct leaching mechanisms: Ca^2+^ leaching is primarily dominated by proton exchange, whereas Si^4+^ leaching is mainly governed by complexation (42). The organic acids produced by the metabolism of strain CMS-1 dissociate into H^+^ and R-COO^−^. The H^+^ is consumed in neutralizing OH^−^, while the R-COO^−^ complexes with Si^4+^, thereby promoting its leaching (43).

In the mixed-culture system, both the leaching efficiency and the degree of enhanced leaching of Ca^2+^ are higher than those of Si^4+^. The primary reasons are as follows: during the bioleaching of wollastonite, Ca^2+^ can be efficiently extracted via acid dissolution by H^+^ and complexation with organic acids. However, the robust Si-O-Si covalent bonds within the structural core [SiO_4_] tetrahedra are difficult to disrupt effectively through protonation alone (44, 45). This fundamentally results in slow leaching kinetics for Si^4+^, leading to its lower final leaching efficiency (18.59%). The observed significantly lower enhancement in Si^4+^ leaching compared to Ca^2+^ indicates the limitations of biological action. Although hyphal attachment and microenvironment acidification can promote the dissolution of the secondary silica gel layer, their contribution to Si^4+^ leaching remains limited. The primary factor is the absence of specific metabolites capable of forming strong soluble complexes with silicic acid, which are required to effectively catalyze Si-O bond cleavage (46). Therefore, the release of Si^4+^ is more constrained by the inherent chemical stability of the siloxane framework, resulting in a relatively modest degree of biological enhancement (47).

Figure 3c shows the variation in pH of the wollastonite leaching suspension across different bioleaching systems. Strain CMS-1 exhibited a relatively weak acid-producing capacity, with the lowest pH reaching approximately 6.9 ± 0.12. In contrast, the addition of strain hzt-3′ significantly enhanced the acidification, reducing the suspension pH to below 4. The period between days 3 and 6 represented the phase of lowest and most stable pH values. The pH in the mixed fungal systems was consistently lower than that in the single-strain systems. Overall, the 20% CMS-1 + 80% hzt-3′ system demonstrated the strongest acid-producing capability and the lowest pH, which consequently ensured the highest Ca^2+^ leaching efficiency.

### Determination of polysaccharide and protein content

**Fig 4:**
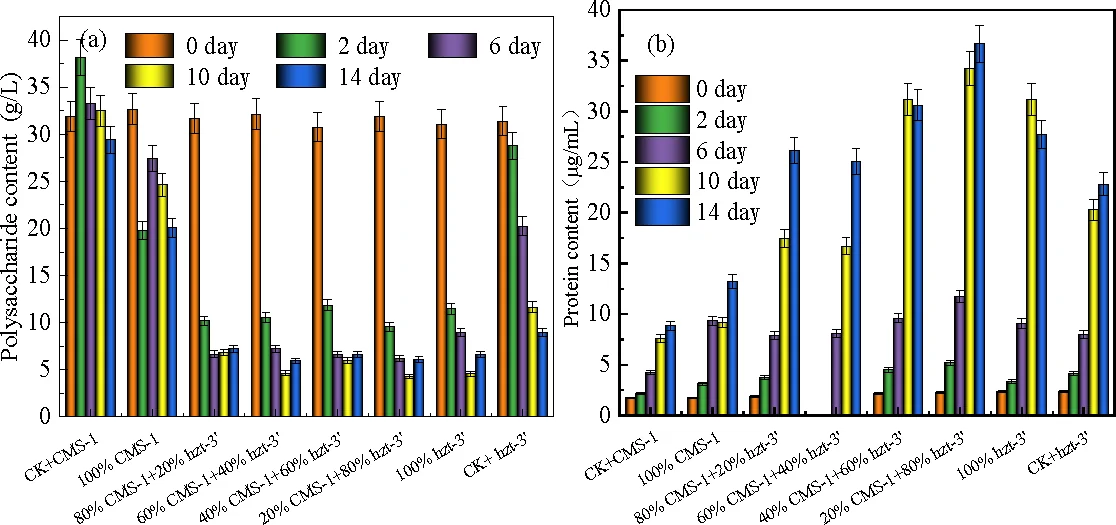
Temporal variations in polysaccharide (**a**) and extracellular protein (**b**) content.

The polysaccharide content in the solution during the bioleaching of wollastonite is shown in Fig. 4a. As can be seen, the polysaccharide content in the 20% CMS-1 + 80% hzt-3′ system was consistently the lowest among all leaching systems. When strain hzt-3′ was present, the polysaccharide content in the bioleaching system gradually decreased as the process proceeded, with the most significant reduction occurring between days 0 and 2. This is attributed to the rapid growth of strain hzt-3′ during this phase, which led to substantial consumption of sucrose from the culture medium, resulting in a marked decrease in polysaccharide content. After day 6, the polysaccharide content remained essentially unchanged, as a dynamic equilibrium was largely maintained between the polysaccharides produced by microbial metabolism and those consumed. In contrast, the increase in polysaccharide content in the pure strain CMS-1 leaching system between days 6 and 10 was due to the higher production of polysaccharides by the strain compared to their metabolic consumption.

Comparing the changes in polysaccharide content in solutions with and without wollastonite reveals a decrease in the bioleaching system containing the mineral. This observation can be explained by two factors: first, the addition of the mineral promoted the consumption of sugar sources by the fungal strains (48); second, the dissolution process of wollastonite itself requires the participation of certain polysaccharides (25).

The extracellular protein content is shown in Fig. 4b. As the proportion of strain hzt-3′ in the mixed culture increased, the extracellular protein content exhibited an initial increase followed by a decrease. The 20% CMS-1 + 80% hzt-3′ system yielded the highest protein content, which contrasted with the trend observed for total sugar. After 14 days of leaching, the extracellular protein content in this system reached 36.67 ± 1.83 µg/mL, which was 2.77 times and 1.32 times higher than that in the 100% CMS-1 (13.21 ± 0.66 µg/mL) and 100% hzt-3′ (27.68 ± 1.38 µg/mL) systems, respectively. When the proportion of hzt-3′ was 60%, 80%, and 100%, the extracellular protein content peaked at day 10, suggesting that fungal metabolic activity likely reached its maximum at this point. In contrast, no distinct peak was observed within the tested period for systems with hzt-3′ proportions of 0%, 20%, and 40%. These results indicated that strain hzt-3′ is a key driver of metabolic activity in the mixed systems, with its proportion requiring a threshold (approximately 50%–60%) to activate metabolism. Increasing its share beyond this threshold effectively accelerates the metabolic activity of the entire leaching process. Further comparison of extracellular protein levels in solutions with and without wollastonite revealed that its presence had minimal impact on the protein content of the pure CMS-1 strain during days 0–2. However, from days 6 to 14, the presence of wollastonite led to an increase in protein content. For strain hzt-3′, the addition of wollastonite had little influence on protein production during days 0–6, but significantly higher protein content was observed in the system containing the mineral from days 10 to 14.

### Determination of organic acid and amino acid content

**Fig 5:**
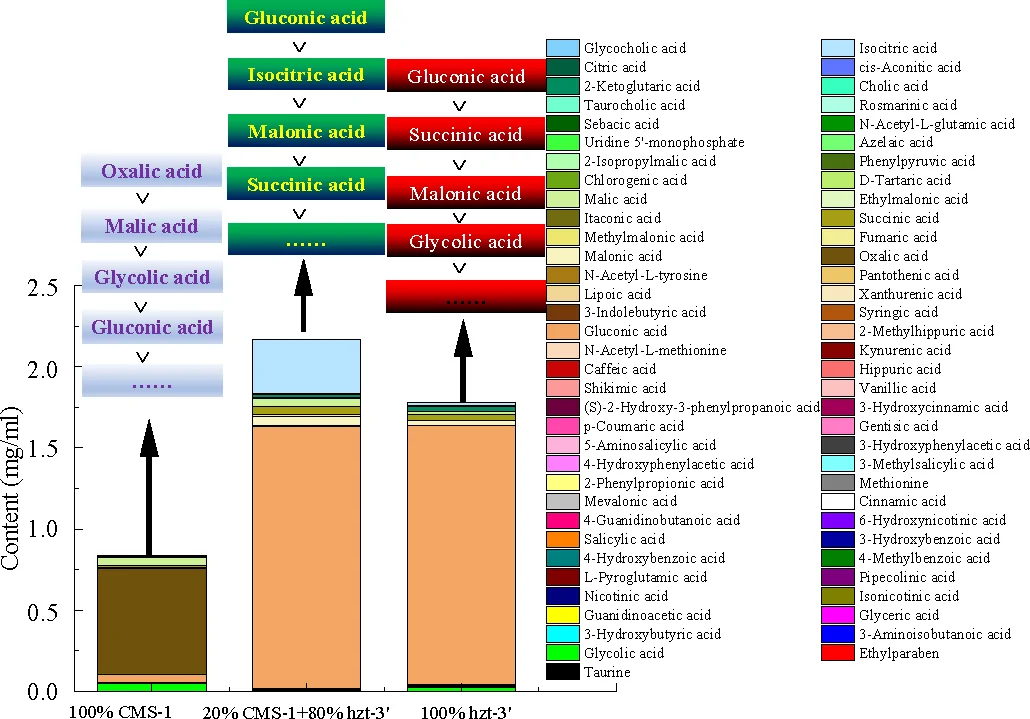
Organic acid content in the wollastonite bioleaching solution.

Figure 5 shows the measured concentrations of organic acids in the wollastonite bioleaching solution, revealing significant differences in both the total amount and the specific types produced across the three systems. The total organic acid production ranked as follows: 20% CMS-1 + 80% hzt-3′ > 100% hzt-3′ > 100% CMS-1. In the 100% CMS-1 system, the primary organic acids and their concentrations were as follows: oxalic acid (0.66 ± 0.033 mg/mL) > malic acid (0.050 ± 0.0022 mg/mL) > glycolic acid (0.046 ± 0.0016 mg/mL) > gluconic acid (0.045 ± 0.0013 mg/mL). The combined concentration of all organic acids other than oxalic acid was substantially lower, indicating that the bioleaching process by strain CMS-1 is predominantly driven by oxalic acid. The production of oxalic acid primarily involves two pathways: (i) isocitrate is converted to glyoxylate by isocitrate lyase, and glyoxylate is then oxidized to oxalate by glyoxylate oxidase or glyoxylate dehydrogenase (49); (ii) oxaloacetate, an intermediate in the tricarboxylic acid cycle, is directly hydrolyzed to oxalate and acetate by oxaloacetate hydrolase (50).

In the pure hzt-3′ strain system, gluconic acid (1.60 ± 0.0798 mg/mL) was the most abundant organic acid, followed by succinic acid (0.033 ± 0.0017 mg/mL), malonic acid (0.031 ± 0.0015 mg/mL), and glycolic acid (0.026 ± 0.013 mg/mL). Gluconic acid, a representative metabolite common to both strains, was produced at approximately 35.35 times higher levels in hzt-3′ than in CMS-1. The mixed fungi system contained four main organic acids, ranked by concentration as: gluconic acid (1.62 ± 0.0811 mg/mL), isocitric acid (0.33 ± 0.0166 mg/mL), malonic acid (0.058 ± 0.0027 mg/mL), and succinic acid (0.051 ± 0.0024 mg/mL). With the exception of isocitric acid, all other representative organic acids were present in both the pure hzt-3′ and mixed fungal systems, indicating that strain hzt-3′ played the dominant role in the mixed culture—a conclusion consistent with the Ca^2+^ leaching results from wollastonite. Notably, the concentration of isocitric acid in the mixed culture was 12.12 times higher than in the pure hzt-3′ system, while its level in the pure CMS-1 system was extremely low (≈3 × 10^−4^ mg/mL). This suggests that the superior leaching performance of the mixed strains may be attributed to the ability of CMS-1 to secrete transcriptases that promote the synthesis of isocitric acid, thereby significantly enhancing its production. The mixed culture also exhibited an extremely low oxalic acid content (only 2.4 × 10^−4^ mg/mL), indicating suppression of its metabolism. This is due to the substantial increase in organic acids, such as gluconic acid, which lowered the system’s pH value. The biosynthetic pathway of oxalic acid—particularly key enzymes like oxaloacetate hydrolase—is highly sensitive to ambient pH value. When the mixed system acidifies due to high gluconic acid production, the metabolic pathway for oxalate synthesis in CMS-1 is inhibited (51). Therefore, the absence of oxalic acid in the mixed fungal system is not due to its decomposition, but rather the cessation of its synthesis.

**Fig 6:**
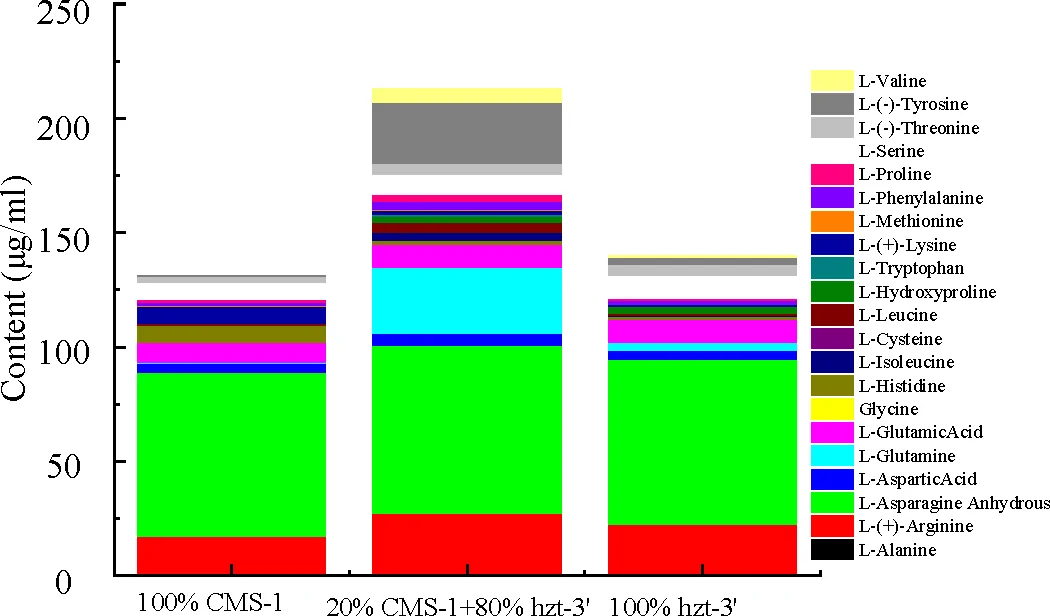
Amino acid content in the wollastonite bioleaching system.


The measured amino acid content is shown in Fig. 6. As illustrated, the mixed-culture system exhibited a significant increase in amino acid levels compared to the pure-culture systems, which is consistent with the extracellular protein measurements (Fig. 6). The differences in amino acid expression across the culture systems were as follows: the concentrations of L-asparagine anhydrous and L-glutamic acid were nearly identical in all three systems. In contrast, the concentration of L-(+)-arginine progressively increased across the 100% CMS-1, 100% hzt-3′, and 20% CMS-1 + 80% hzt-3′ systems, reaching 17.32 ± 0.87 µg/mL, 22.65 ± 1.08 µg/mL, and 27.35 ± 1.33 µg/mL, respectively. The expression of L-glutamine was highest in the mixed-culture system (28.99 ± 1.44 µg/mL) and lowest in the 100% CMS-1 pure culture (0.56 ± 0.028 µg/mL), representing an approximately 51.77-fold difference. This significant discrepancy can be attributed to the fact that strain CMS-1 can produce a substantial amount of L-glutamic acid (9.63 ± 0.46 µg/mL) from the substrate; however, due to its own regulatory mechanisms, its glutamine synthetase is either deficient or exhibits low activity, making it difficult to synthesize L-glutamine (52). In contrast, while no L-glutamic acid was detected in the strain hzt-3′ solution, the expression level of L-glutamine was 9.26 ± 0.46 µg/mL, indicating high GS activity that completely converted L-glutamic acid into L-glutamine (52). When the two strains were mixed, the L-glutamic acid and ATP produced by strain CMS-1 were efficiently utilized by strain hzt-3′ to synthesize glutamine. This “division of labor” resulted in overall system productivity and yield that exceeded the levels achieved by either strain in pure culture. The reaction for the synthesis of L-glutamine from L-glutamic acid is shown in equation 3 (53).

(3) $$L\text{-}glutamic\;acid+NH_{3}+ATP\overset{GS}{\to }L\text{-}glutamine+ADP+P\text{i}$$

In addition to L-glutamine, the expression level of L-(-)-tyrosine also exhibited substantial differences across the systems, with concentrations of 0.80 ± 0.035 µg/mL in 100% CMS-1, 2.71 ± 0.13 µg/mL in 100% hzt-3′, and 26.39 ± 1.22 µg/mL in the 20% CMS-1 + 80% hzt-3′ mixed culture. This represents up to a 32.99-fold increase in the mixed culture compared to the pure cultures. This phenomenon can be attributed to the altered population density in the mixed-culture system, which may trigger quorum-sensing mechanisms that are not activated in pure cultures (54). These signaling molecules could potentially upregulate key genes in the L-(-)-tyrosine biosynthesis pathway or downregulate competing pathways, thereby redirecting carbon flux toward enhanced L-(-)-tyrosine synthesis.

### Analysis of the physicochemical properties of the leaching residues

**Fig 7:**
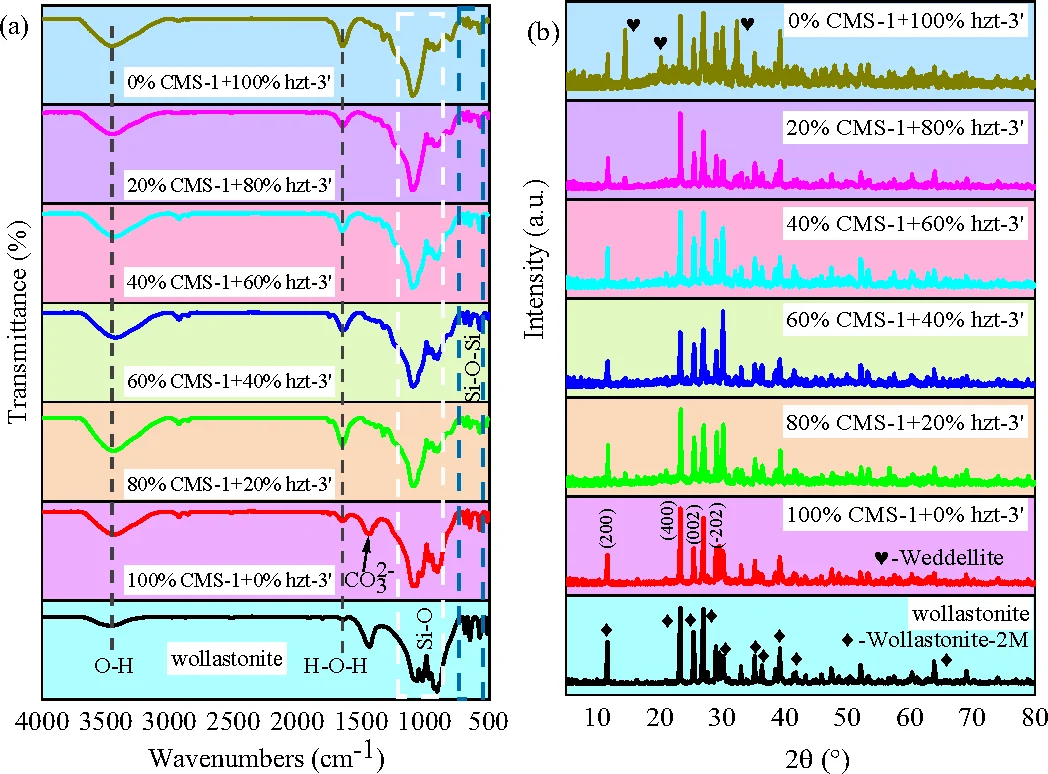
FT-IR (a) and phase analysis (b) of wollastonite and its bioleaching residues.

Figure 7a shows the FT-IR results of raw wollastonite and the leaching residues. As a single-chain silicate mineral with the chemical formula CaSiO_3_, wollastonite lacks hydroxyl (-OH) groups in its crystal structure; consequently, its infrared spectrum is dominated by Si-O bond vibrations, with characteristic peaks primarily appearing in two regions: Si-O stretching vibration (1,100–900 cm^−1^) and Si-O-Si bending vibration (700–400 cm^−1^) (55, 56). Specifically, the shoulder at 1,043 cm^−1^ is assigned to the asymmetric stretching vibration of the Si-O-Si bridging bond, while the strongest peak at 982 cm^−1^—the main peak of wollastonite—is attributed to the stretching vibration of the intra-chain Si-O^−^ (non-bridging oxygen) and serves as a key identifier for the mineral. The characteristic peaks at 900 and 942 cm^−1^ are also attributed to Si-O^−^ stretching vibrations. The peak at 1,428 cm^−1^ corresponds to the asymmetric stretching (v₃) of carbonate ions (CO_3_^2−^) (57), which almost completely disappeared in the leaching residues of systems containing strain hzt-3′ but remained present in the pure CMS-1 system. This observation is consistent with the generally higher calcium ion concentration in cultures containing hzt-3′. In the low wavenumber range, the peaks at 500 and 564 cm^−1^ are assigned to O-Si-O bending vibrations, while those at 639 and 681 cm^−1^ are attributed to Si-O-Si symmetric stretching/bending vibrations (57). In the leaching residues, the peak at 1,043 cm^−1^ completely disappeared, and a characteristic absorption peak of amorphous silica (SiO_2_) emerged at 1,086 cm^−1^, attributed to the asymmetric stretching vibration of the Si-O-Si bond (58). This indicated that microbial activity led to the destruction of the wollastonite crystal structure and the formation of an amorphous silica phase. These changes were most pronounced in the 20% CMS-1 + 80% hzt-3′ mixed-culture system, corresponding to the highest Ca^2+^ leaching efficiency observed in this system during ion concentration measurements.

The phase composition of wollastonite is shown in Fig. 7b. The raw ore was primarily composed of wollastonite-2M (PDF#27-0088). A slight decrease in the intensity of the corresponding characteristic peaks was observed in the leaching residues, with the 20% CMS-1 + 80% hzt-3′ system showing the most notable reduction. In the residue from the pure hzt-3′ culture system, characteristic peaks of weddellite (PDF#17-0541) appeared at 2*θ* = 14.32°, 20.07°, and 32.23°, indicating the formation of the secondary mineral calcium oxalate dihydrate. Overall, the minimal phase change in wollastonite before and after fungal treatment can be attributed to the leaching reaction primarily occurring at the surface and interfaces of the mineral particles. A significant portion of the crystal interior may have remained unreacted or undergone only superficial reaction, meaning the XRD signals predominantly originated from the unreacted or slightly reacted crystal cores, resulting in negligible shifts in peak positions and intensity. Nevertheless, the significant increase in Ca^2+^ and Si^4+^ concentrations in the leaching solution provides direct evidence that the fungal leaching process did occur.

The interplanar spacing for the four diffraction peaks that showed the most pronounced changes in the XRD patterns was calculated, and the results are presented in Table 2. After fungal treatment, the four main diffraction peaks of the bioleaching residues shifted to higher angles, indicating a reduction in interplanar spacing. This is because during the bioleaching process, H^+^ generated from the ionization of organic acids secreted by the fungi exchange with Ca^2+^ in the crystal structure of wollastonite. This ion exchange alters the atomic arrangement within the wollastonite lattice. To maintain lattice equilibrium, the interatomic distances shorten, leading to lattice contraction (37). Compared to other bioleaching systems, the wollastonite residues treated with the 20% CMS-1 + 80% hzt-3′ co-culture showed the most pronounced shift in diffraction peak positions (2*θ*), the greatest loss in structural order, the largest reduction in interplanar spacing (*d*), and the most severe disruption of the crystal structure. These structural changes align with the highest leaching efficiencies observed for both Ca^2+^ and Si^4+^.

**TABLE 2:** Interplanar spacings (*d*) and corresponding 2*θ* values of diffraction peaks indexed by Miller indices

Samples	(200)		(400)		(002)		(−202)	
	2θ/°	*d*/Å	2θ/°	*d*/Å	2θ/°	*d*/Å	2θ/°	*d*/Å
Wollastonite	11.620	7.609	23.276	3.819	25.345	3.511	26.970	3.303
100% CMS-1 + 0% hzt-3′	11.650	7.590	23.280	3.818	25.420	3.501	26.975	3.303
80% CMS-1 + 20% hzt-3′	11.700	7.558	23.325	3.811	25.443	3.498	27.020	3.297
60% CMS-1 + 40% hzt-3′	11.703	7.556	23.298	3.815	25.462	3.495	26.980	3.302
40% CMS-1 + 60% hzt-3′	11.684	7.568	23.321	3.811	25.480	3.493	27.010	3.299
20% CMS-1 + 80% hzt-3′	11.719	7.545	23.332	3.809	25.513	3.489	27.019	3.297
0% CMS-1 + 100% hzt-3′	11.715	7.548	23.294	3.816	25.481	3.493	26.983	3.302

**Fig 8:**
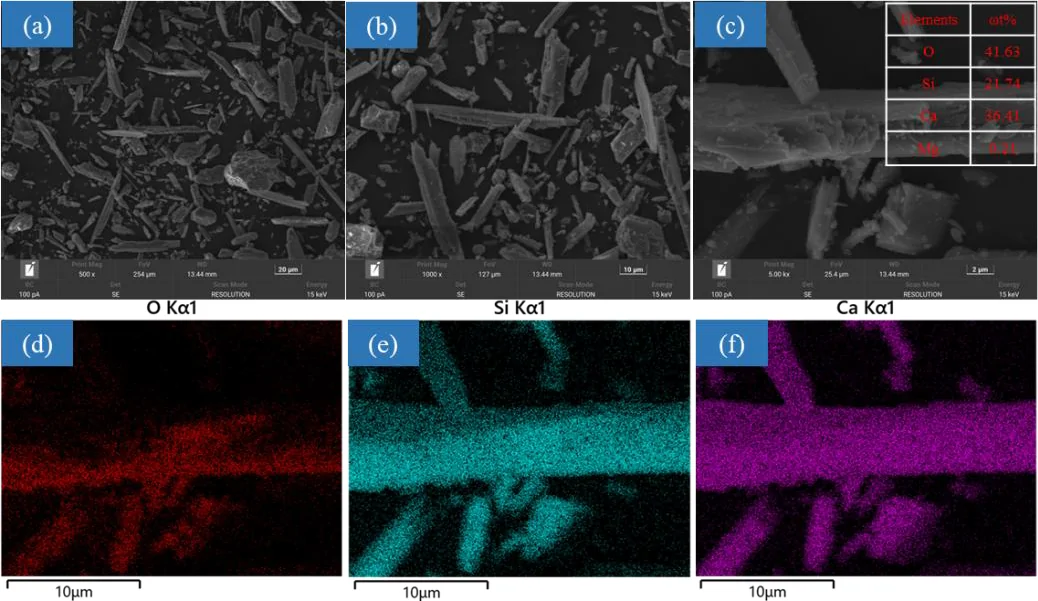
Morphology and elemental distribution of wollastonite: (**a**, **b**, and **c**) microtopography; (**d**) O element distribution; (**e**) Si element distribution; (**f**) Ca element distribution.

The morphological characteristics and elemental distribution of the wollastonite sample were characterized by SEM and EDS, with the results shown in Fig. 8. As seen in Fig. 8a through c, the crystal morphology of wollastonite is predominantly acicular and columnar, accompanied by a minor amount of flaky structure, with an uneven size distribution ranging mostly from several to tens of micrometers. The crystal surfaces are smooth, exhibiting good crystallinity, distinct cleavage planes, and well-defined edges. EDS mapping results indicated that oxygen (red) is widely distributed throughout the wollastonite regions (Fig. 8d), consistent with the structural characteristics of silicate minerals; silicon (cyan) showed a distribution highly coincident with oxygen, primarily concentrated within the acicular and columnar crystals (Fig. 8e), confirming the presence of a SiO_4_ tetrahedral framework. Calcium (purple) was also enriched in the crystal areas, though its distribution intensity exhibits slight local fluctuations (Fig. 8f), possibly due to minor substitutions or defects. The measured Ca/Si ratio in wollastonite is 1.67, which is slightly higher than the theoretical value for CaSiO_3_ (Ca/Si = 1.43) (59), potentially attributable to surface adsorption or trace calcium enrichment, yet overall consistent with the stoichiometric characteristics of wollastonite.

**Fig 9:**
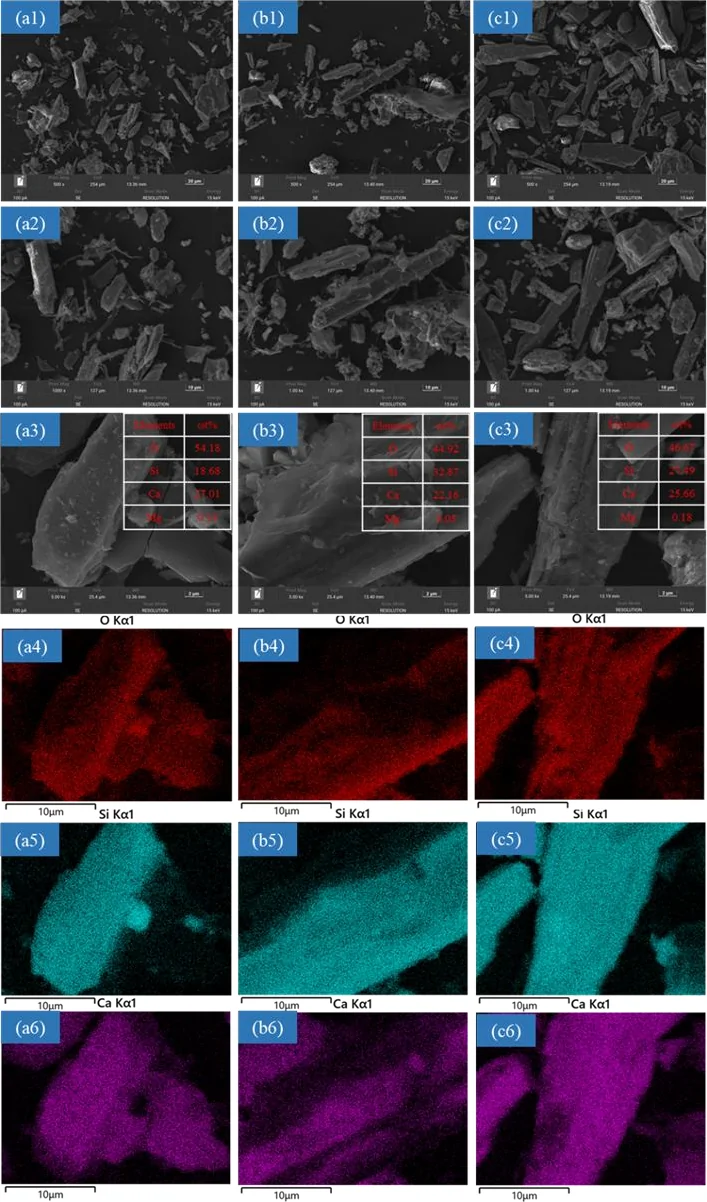
SEM images and elemental maps of the bioleaching residues: (**a**) 100% CMS-1, (**b**) 20% CMS-1 + 80% hzt-3′, and (**c**) 100% hzt-3′.

Figure 9 shows the morphological characteristics and elemental distribution of wollastonite leaching residues treated by the three culture systems. In the 100% CMS-1 system, the residues primarily consisted of irregular blocks with partially retained acicular structures, exhibiting erosion grooves and cracks on the surface (Fig. 9a1 through a3), indicating limited dissolution. In contrast, the 20% CMS-1 + 80% hzt-3′ system displayed the most significant morphological changes, where the original crystal structure was completely disrupted, forming loose, porous, flocculent aggregates (Fig. 9b1 through b3). This morphology suggests that the organic acids produced by both strains caused severe corrosion of the mineral crystals. The residues from the 100% hzt-3′ system appeared as fragmented particles of varying sizes with rough surfaces, exhibiting an overall degree of corrosion intermediate between the 100% CMS-1 and the 20% CMS-1 + 80% hzt-3′ systems. Integrating these morphological observations, it can be concluded that the 20% CMS-1 + 80% hzt-3′ mixed culture had the most significant bioleaching effect on wollastonite, disrupting its crystal structure through microbe-mineral interactions.

Based on the EDS elemental analysis results, the residues primarily consisted of O, Si, and Ca. Oxygen (red) was uniformly distributed across the entire observed area (Fig. 9a4 through c4). The silicon signal (cyan) was also relatively widespread and evenly distributed (Fig. 9a5 through c5), indicating the relatively slow dissolution of SiO_4_ tetrahedra or their partial retention in the residues as amorphous silica (SiO_4_·nH_2_O). The distribution trend of calcium directly corresponded to the leaching effectiveness: strong Ca signals in Fig. 9a6 and c6 correspond to residual mineral phases, whereas the significantly weakened and discrete Ca signal in Fig. 9b6 indicates substantial dissolution and migration of calcium. Calculation of the mass Ca/Si ratio in the residues yielded values of 1.44 for group a, 0.67 for group b, and 0.93 for group c, all lower than the value in pristine wollastonite (Ca/Si = 1.67). The most pronounced decrease was observed in group b (the 20% CMS-1 + 80% hzt-3′ culture system). This result demonstrates that the mixed-strain leaching achieved the highest efficiency of selective calcium leaching, leading to silicon enrichment and the lowest Ca/Si ratio in the residue. This finding corroborates the observation of the most severe corrosion in the morphological analysis, confirming the superior bioleaching effectiveness of the mixed strains on wollastonite.

**Fig 10:**
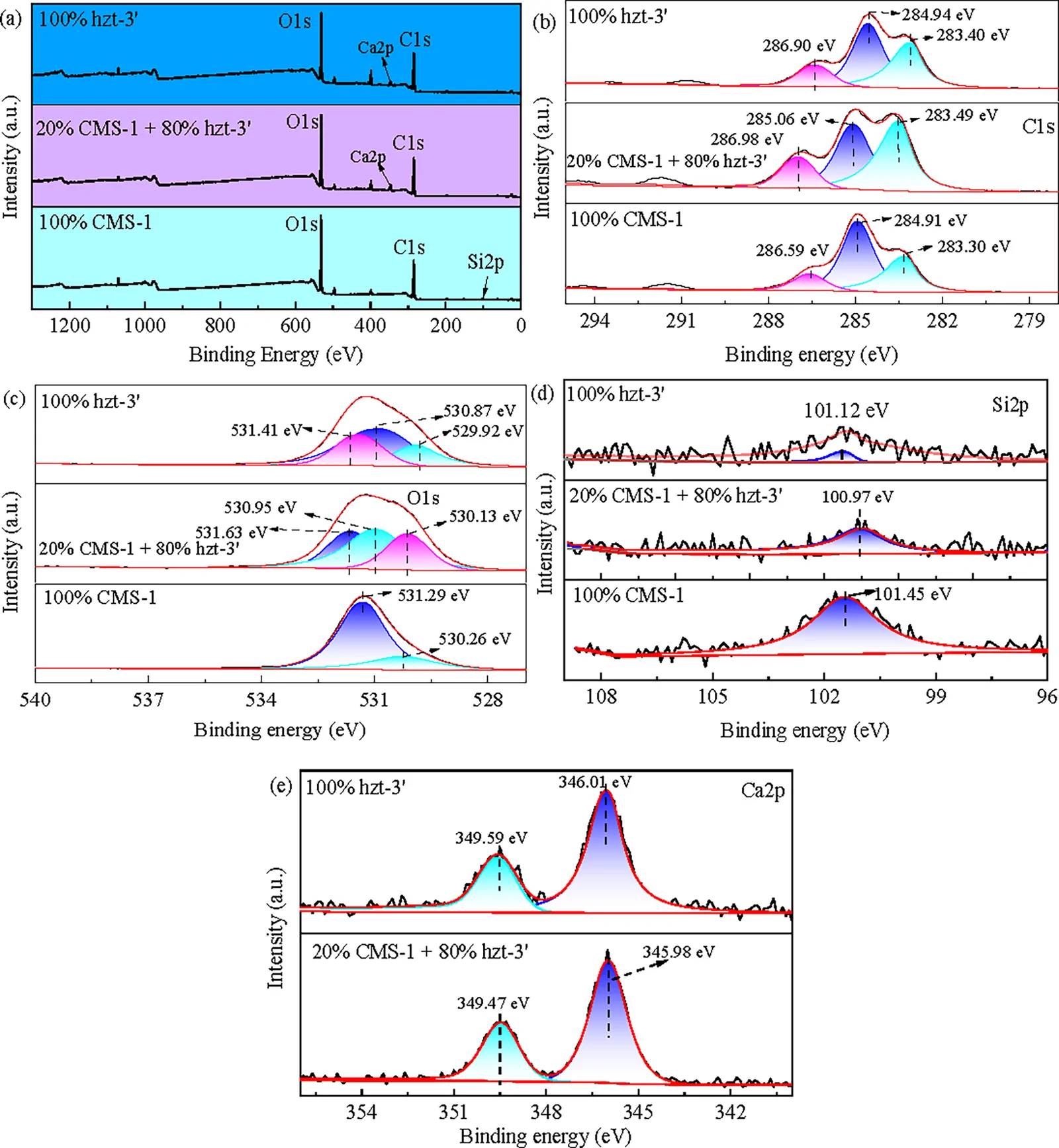
XPS spectra of fungal broth: (**a**) survey spectrum, (**b**) C 1s, (**c**) O 1s, (**d**) Si 2p, and (**e**) Ca 2p.

The XPS results of elements in the fungal broth are shown in Fig. 10. In the 100% CMS-1 culture system, no Ca signal was detected (Fig. 10a), which is consistent with the extremely low Ca^2+^ concentration observed in the leaching results. The peak in the C 1s spectrum at 286.98 eV is attributed to C-O bonds (Fig. 10c), primarily originating from hydroxyl or carboxyl groups in the organic acids secreted by the fungi. This serves as direct evidence that the fungi promote mineral leaching through the secretion of organic acids (60). The relative contribution of carbon from C-O bonds of organic functional groups is more significant in the mixed culture, indicating that the mixed microbial community produces a greater quantity of metabolites such as organic acids. The O 1s peak at ~530.13 eV is typically assigned to lattice oxygen in wollastonite, the peak at ~530.95 eV is attributed to oxygen in Si-O bonds or carboxyl groups (C=O) of organic acids, while the peak near ~531.63 eV corresponds to adsorbed water or hydroxyl groups (-OH) (60, 61). In the mixed culture, the intensity of the peak representing lattice oxygen shows an increasing trend, possibly suggesting a slightly enhanced disruption of the mineral lattice by the mixed community, releasing more lattice oxygen. The Si 2p binding energy is near 101.45 eV (Fig. 10d), which falls within the typical binding energy range for monomeric silicic acid or silicate Si^4+^ (62, 63). This indicates that silicon in the leachate mainly exists in the form of dissolved calcium silicate complexes or silicic acid (H_4_SiO_4_). The Ca 2p spectra of pure and mixed cultures are highly similar (Fig. 10e), indicating the presence of calcium as dissolved calcium silicate complexes or free Ca^2+^ (64).

No Ca 2p signal was detected in the XPS spectra of the fungal mycelia (Fig. S5), indicating efficient leaching of calcium without residual deposition on the mycelial surfaces. The mycelia only enrich the leached Si^4+^. The Si 2p binding energy of the mycelia remains stable within the range of approximately 101.40–101.53 eV (Fig. S5d), closely matching the typical binding energy of organosilicate or monomeric silicic acid (62, 63). This suggests that the silicon present on the mycelial surfaces originates from dissolved wollastonite residues or silicon species that have been re-adsorbed or precipitated onto the mycelia in the form of silicic acid/silicate.

**Fig 11:**
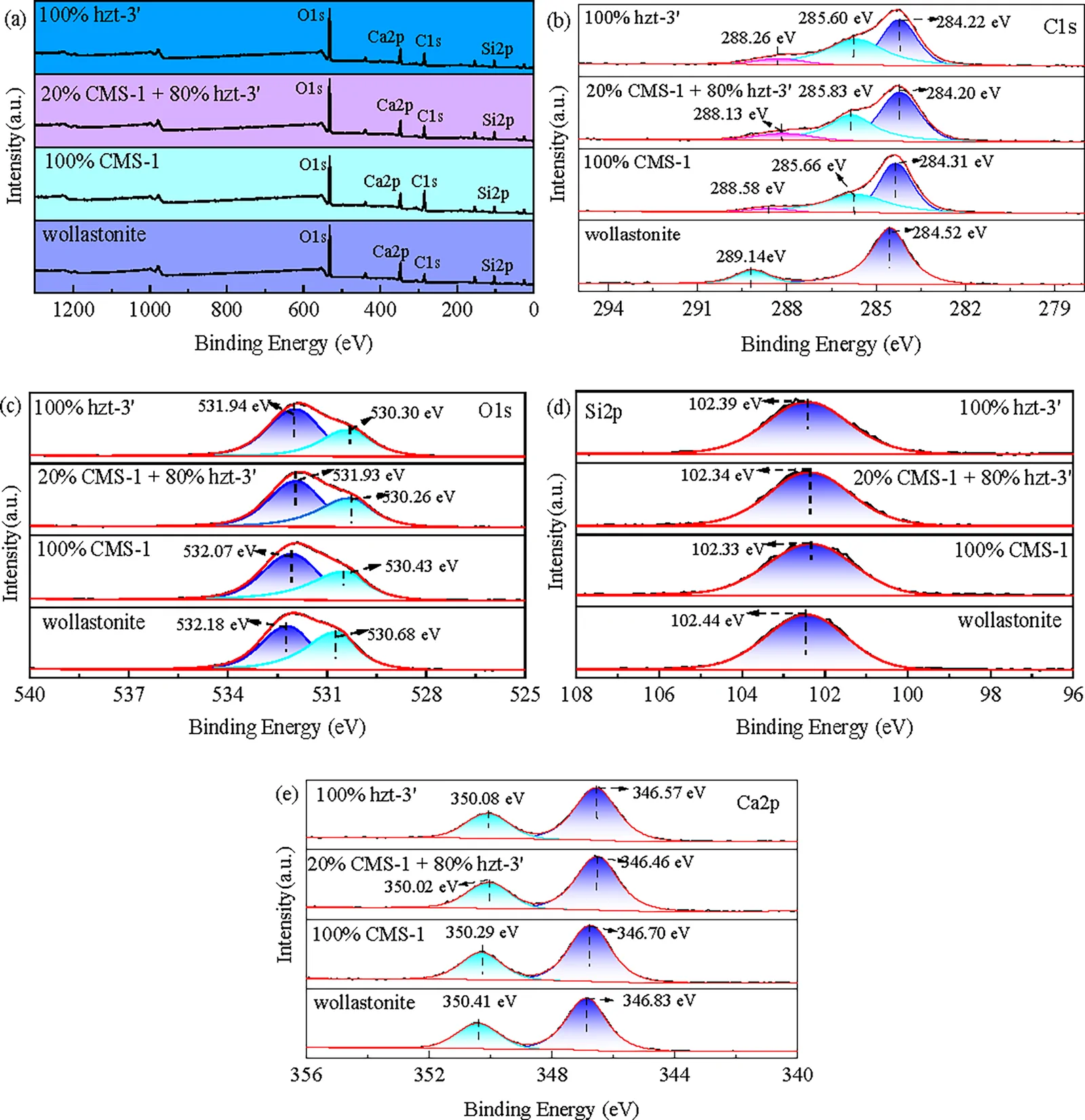
XPS spectra of wollastonite and bioleaching residues: (**a**) survey spectrum, (**b**) C 1s, (**c**) O 1s, (**d**) Si 2p, and (**e**) Ca 2p.

Figure 11 shows the XPS spectra of wollastonite and bioleaching residues. In the full survey scan (Fig. 11a), the C 1s peak of pristine wollastonite is relatively weak, whereas the intensity and proportion of the C 1s peak increase dramatically in the bioleaching residues, indicating the attachment of fungal mycelia, extracellular polymeric substances, and metabolites or the formation of a biofilm on the mineral surface. The relative intensities of the Ca 2p and Si 2p peaks in the residues generally decrease, which can be attributed to heterogeneous dissolution of the mineral surface or partial shielding of the characteristic mineral signals by the covering layer of biogenic organic matter. After bioleaching, the binding energies of O 1s and Si 2p show only a slight negative shift (Fig. 11c and d), with their peak shapes remaining largely unchanged. This suggests that during fungal leaching, the primary attack is on the Ca-O bonds in the mineral, while the silicate tetrahedral framework (Si-O) largely retains its original chemical structure at the mineral surface. A negative shift in the Ca 2p binding energy is observed in the bioleaching residues (Fig. 11e), accompanied by a significant enhancement of the C=O/O-C=O peak (~288.13 eV) in the C 1s spectrum (Fig. 11b). This indicates that some of the Ca^2+^ leached from the lattice have undergone complexation with organic acids secreted by the fungi, forming calcium–organic acid complexes on the mineral surface, which increases the electron density around Ca and consequently lowers its binding energy (64, 65). This phenomenon is more pronounced in the mixed-culture system, suggesting that the synergistic metabolism of the mixed microbial community leads to higher efficiency in the complexation and leaching of calcium compared to single-strain cultures.

### Enhanced element leaching mechanism by mixed fungi

**Fig 12:**
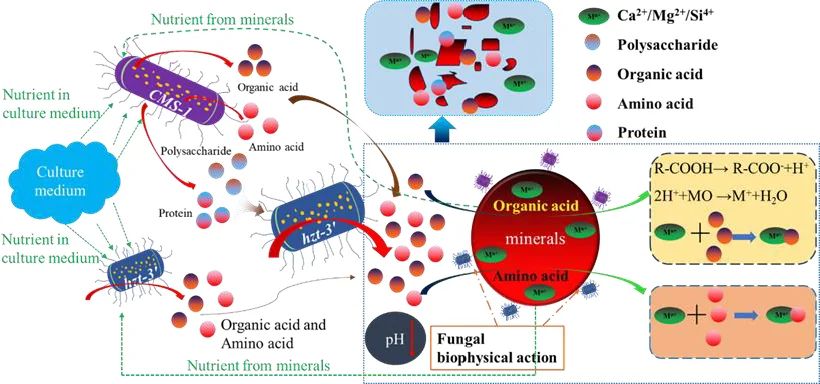
Mechanism of enhanced element leaching by the mixed fungal culture.

The mechanism of enhanced element leaching from wollastonite by the mixed fungal culture is illustrated in Fig. 12. A synergistic effect, characterized by metabolic complementarity and niche differentiation, exists between the strains during mixed cultivation. The strain CMS-1 acts as a “primary producer,” utilizing sucrose from the culture medium to produce polysaccharides, extracellular proteins, organic acids, and amino acids. The strain hzt-3′ functions as a “potent acid producer.” Upon acquiring the extracellular polysaccharides and proteins provided by CMS-1, the growth and metabolic activity of hzt-3′ are significantly enhanced, leading to a substantial increase in the production of organic acids (e.g., gluconic acid and isocitric acid) and amino acids (e.g., L-glutamine and L-tyrosine). This results in an overall increase in the acidity of the mixed system, a decrease in solution pH, and consequently, an enhanced corrosion capacity of the solution.

Chemical formula for gluconic acid dissolution and complexation is as follows (66):


(4) $${2HC}_{6}H_{11}O_{7}\to 2C_{6}H_{11}O_{7}^{-}+2H^{+},$$

(5) $${CaSiO}_{3}+2H^{+}\to {Ca}^{2+}+H_{2}{SiO}_{3},$$

(6) $$2[C_{6}H_{11}O_{7}^{-}]+{Ca}^{2+}\to Ca[C_{6}H_{11}O_{7}{]}_{2}.$$

The equations for the acid dissolution and complexation reactions of isocitric acid are as follows (67, 68):


(7) $$H_{3}C_{6}H_{5}O_{7}\to H_{2}C_{6}H_{5}O_{7}^{-}+H^{+},$$

(8) $$H_{2}C_{6}H_{5}O_{7}^{-}\to {HC}_{6}H_{5}O_{7}^{2-}+H^{+},$$

(9) $${HC}_{6}H_{5}O_{7}^{2-}\to C_{6}H_{5}O_{7}^{3-}+H^{+},$$

(10) $${CaSiO}_{3}+2H^{+}\to {Ca}^{2+}+H_{2}{SiO}_{3},$$

(11) $$2[H_{2}C_{6}H_{5}O_{7}^{-}]+{Ca}^{2+}\to Ca[H_{2}C_{6}H_{5}O_{7}{]}_{2},$$

(12) $$[{HC}_{6}H_{5}O_{7}^{2-}]+{Ca}^{2+}\to Ca[{HC}_{6}H_{5}O_{7}],$$

(13) $$2[C_{6}H_{5}O_{7}^{3-}]+3{Ca}^{2+}\to {Ca}_{3}[C_{6}H_{5}O_{7}{]}_{2}.$$

The carboxyl groups (-COOH) in organic acids provide H^+^ for acidic dissolution, breaking the Ca-O bonds in the wollastonite crystal structure. Simultaneously, they act as coordination sites, forming stable chelates with Ca^2+^ to prevent the precipitation of dissolved Ca^2+^. The presence of hydroxyl groups (-OH) enhances the water solubility of organic acid molecules and may also participate in coordination, increasing the stability of the complexes (42).

Due to the relatively weak acidity of amino acid carboxyl groups, their primary mechanism is complexation rather than acidolysis (69). Amino acid molecules act as ligands, forming soluble complexes with Ca^2+^ on the wollastonite surface, thereby enhancing the release of calcium ions from the mineral lattice. The chemical equations for wollastonite leaching by L-glutamine and L-tyrosine are shown in equations 14 and 15, respectively:


(14) $${CaSiO}_{3}+2C_{5}H_{9}N_{2}O_{3}\to Ca(C_{5}H_{8}N_{2}O_{3}{)}_{2}+H_{2}{SiO}_{3},$$

(15) $${CaSiO}_{3}+2C_{9}H_{11}{NO}_{3}\to Ca(C_{9}H_{10}{NO}_{3}{)}_{2}+H_{2}{SiO}_{3}.$$

The H^+^ dissociated from the carboxyl groups (-COOH) in amino acids can weakly attack the mineral surface, while the negatively charged (-COO^−^) groups adsorb Ca^2+^ through electrostatic interactions, preparing for subsequent coordination. The nitrogen atoms on the amino groups (-NH_2_) of amino acids possess lone pairs of electrons, enabling them to form stable chelates with Ca^2+^ (69). This strong chelation significantly reduces the concentration of free Ca²^+^ in solution, driving the reaction forward—a key distinction from the action of organic acids.

The increased levels of both organic acids and amino acids in the mixed-culture system undoubtedly intensify these chemical effects. Beyond the aforementioned chemical interactions, the fungi also exert biophysical effects during their interaction with the mineral. Fungal hyphae extend via apical growth, penetrating into mineral cracks and pores, thereby disrupting the crystal structure and accelerating the release of elements from the mineral (28).

### Conclusions

This study conducted a detailed investigation into the leaching patterns of Ca^2+^ and Si^4+^ from wollastonite and the synergistic mechanisms between the fungi within a mixed-culture system of *Ramichloridium apiculatum* CMS-1 and *Talaromyces pseudofuniculosus* hzt-3′. The key findings are summarized as follows:

1. The leaching efficiency of the mixed-strain system was significantly superior to that of the pure culture. The 20% CMS-1 + 80% hzt-3′ system demonstrated the most effective dissolution. Compared to the pure systems of 100% CMS-1 and 100% hzt-3′, the Ca^2+^ leaching rate was enhanced by 30.17 times and 1.11 times, respectively, while the Si^4+^ leaching efficiency was increased by 1.42 times and 1.30 times, respectively.
2. During mixed cultivation, the altered population density may involve quorum-sensing mechanisms that are not activated in pure culture. This led to more vigorous metabolic activity within the microbial community, accelerating the consumption of polysaccharides in the system. Consequently, it resulted in increased extracellular protein content and a decrease in the pH of the solution environment.
3. A synergistic effect enhances wollastonite bioleaching by the mixed strains. CMS-1 acts as a “primary producer,” metabolizing sucrose into extracellular polysaccharides and proteins. Strain hzt-3′, a “potent acid producer,” utilizes these metabolites to significantly increase organic acid (e.g., gluconic acid and isocitric acid) and amino acid (e.g., L-glutamine and L-tyrosine) secretion, thereby elevating system acidity and enhancing mineral dissolution. Furthermore, fungal hyphae exert direct biophysical disruption on the mineral.

## SUPPLEMENTAL MATERIAL

Supplemental material aem.02519-25-s0001.docx Fig. S1 to S5.

ASM does not own the copyrights to Supplemental Material that may be linked to, or accessed through, an article. The authors have granted ASM a non-exclusive, world-wide license to publish the Supplemental Material files. Please contact the corresponding author directly for reuse.
