# Supplementary material for: Effect and mechanism of mixed fungi synergistically enhancing the leaching of elements from wollastonite
Source: Appl Environ Microbiol. 2026 Jul 21;92(8):e02519-25. doi: 10.1128/aem.02519-25 (PMC13488302; doi:10.1128/aem.02519-25)
Supplement: Supplementary file 1 [file aem.02519-25-s0001.docx]

*Supporting Information for*

Effect and Mechanism of Mixed Fungal Synergistically Enhancing the Elements Leaching from Wollastonite

Chengbing Chang^1^, Jianying Guo^1^, Shengyu Liu^1,2*^, Quanbao Wen^1^

1 College of Mining Engineering, Taiyuan University of Technology, Taiyuan, Shanxi 030024, China

2 Key Laboratory of In-situ Property-improving Mining of Ministry of Education, Taiyuan University of Technology, Taiyuan 030024, Shanxi, China

*Corresponding author: Shengyu Liu, Email: [liusytyut@sina.com](mailto:liusytyut@sina.com)

This supporting information contains 5 Figures

After conducting a BLAST alignment of the ITS sequence of strain CMS-1 and hzt-3' in the NCBI database, a phylogenetic tree was constructed using the neighbor-joining method in MEGA 11.0 software, as shown in Fig S1 and Fig S2. The ITS sequencing results showed that the similarity between fungal CMS-1 and *Ramichloridium apiculatum* was 100%, which was further confirmed in the phylogenetic tree. Strain hzt-3' exhibited a homology of 99.78% with *Talaromyces pseudofuniculosus* NBRC 172396. Combined with the phylogenetic evolutionary tree analysis, this further confirms that strain hzt-3' is identified as *Talaromyces pseudofuniculosus*.

Fig. S1 Phylogenetic tree of strain CMS-1

Fig. S2 Phylogenetic tree of strain hzt-3' based on ITS gene sequences

Fig. S3 Standard curve for polysaccharide content determination

Fig. S4 Standard curve for protein content determination

Fig. S5 XPS spectra of fungal mycelium: (a) Survey spectrum, (b) C 1s, (c) O 1s, (d) Si 2p
